# Biofilm Matrix Composition Affects the Susceptibility of Food Associated Staphylococci to Cleaning and Disinfection Agents

**DOI:** 10.3389/fmicb.2016.00856

**Published:** 2016-06-06

**Authors:** Annette Fagerlund, Solveig Langsrud, Even Heir, Maria I. Mikkelsen, Trond Møretrø

**Affiliations:** ^1^Nofima, Norwegian Institute of Food, Fisheries and Aquaculture ResearchÅs, Norway; ^2^Department of Chemistry, Biotechnology and Food Science, Norwegian University of Life SciencesÅs, Norway

**Keywords:** *Staphylococcus*, biofilm, matrix, ica, MSCRAMM, quaternary ammonium compound, benzalkonium chloride

## Abstract

Staphylococci are frequently isolated from food processing environments, and it has been speculated whether survival after cleaning and disinfection with benzalkonium chloride (BC)-containing disinfectants is due to biofilm formation, matrix composition, or BC eﬄux mechanisms. Out of 35 food associated staphylococci, eight produced biofilm in a microtiter plate assay and were identified as *Staphylococcus capitis* (2), *S. cohnii*, *S. epidermidis*, *S. lentus* (2), and *S. saprophyticus* (2). The eight biofilm producing strains were characterized using whole genome sequencing. Three of these strains contained the *ica* operon responsible for production of a polysaccharide matrix, and formed a biofilm which was detached upon exposure to the polysaccharide degrading enzyme Dispersin B, but not Proteinase K or trypsin. These strains were more tolerant to the lethal effect of BC both in suspension and biofilm than the remaining five biofilm producing strains. The five BC susceptible strains were characterized by lack of the *ica* operon, and their biofilms were detached by Proteinase K or trypsin, but not Dispersin B, indicating that proteins were major structural components of their biofilm matrix. Several novel cell wall anchored repeat domain proteins with domain structures similar to that of MSCRAMM adhesins were identified in the genomes of these strains, potentially representing novel mechanisms of *ica*-independent biofilm accumulation. Biofilms from all strains showed similar levels of detachment after exposure to alkaline chlorine, which is used for cleaning in the food industry. Strains with *qac* genes encoding BC eﬄux pumps could grow at higher concentrations of BC than strains without these genes, but no differences were observed at biocidal concentrations. In conclusion, the biofilm matrix of food associated staphylococci varies with respect to protein or polysaccharide nature, and this may affect the sensitivity toward a commonly used disinfectant.

## Introduction

Despite daily cleaning and disinfection, staphylococci are frequently isolated from machines and surfaces in food processing plants (Sundheim et al., 1992; Møretrø et al., 2003; Marino et al., 2011). Coagulase negative staphylococci (CNS) dominate, but also the food borne pathogen *Staphylococcus aureus* that may cause intoxications in humans and mastitis in cows has been isolated from food processing environments (Langsrud et al., 2006; Marino et al., 2011). Survival of staphylococci in the harsh conditions may be linked biofilm formation protecting them from detachment by cleaning agents and killing by disinfectants and specific resistance mechanisms such as eﬄux pumps (Campanac et al., 2002; Luppens et al., 2002; Wassenaar et al., 2015).

Biofilms of staphylococci are common sources of infections on medical implants in the human body (Arciola et al., 2015), and the mechanisms of biofilm formation have been studied in detail for clinical *S. aureus* and *S. epidermidis.* The most common mechanism of biofilm formation in these species depends on production of the polysaccharide intercellular adhesin (PIA) as the most important component of the biofilm matrix. PIA is produced by the proteins encoded by the *ica* operon comprising the *icaADBC* genes and the regulatory gene *icaR* (Arciola et al., 2015). Extracellular DNA (eDNA) and cell wall associated teichoic acids are also believed to have structural roles in *S. aureus* and *S. epidermidis* biofilms, while unspecific electrostatic and hydrophobic interactions mediated by teichoic acids, eDNA, and hydrophobic surface proteins can contribute to primary adhesion to abiotic surfaces (Izano et al., 2008; Jabbouri and Sadovskaya, 2010; Becker et al., 2014; Büttner et al., 2015).

*Staphylococcus aureus* and *S. epidermidis* strains that can produce biofilms without PIA exopolysaccharide are dependent on protein-mediated intercellular adhesion. It is recognized that several staphylococcal cell wall anchored (CWA) surface proteins may promote not only surface adhesion to biotic and abiotic surfaces, but also the accumulation phase of biofilm formation through mediating cell–cell adhesion (Foster et al., 2014; Speziale et al., 2014; Arciola et al., 2015). These include SdrC, ClfB, FnBPA, and FnBPB, which belong to the class of CWA proteins originally termed microbial surface components recognizing adhesive matrix molecules (MSCRAMM) based on their ability to mediate specific interaction with components of human extracellular matrix (ECM; Abraham and Jefferson, 2012; Geoghegan et al., 2013; Barbu et al., 2014). MSCRAMMs are characterized by having a non-repetitive N-terminal adhesion domain composed of two or three immunoglobulin (IgG)-like folds, followed by a region of tandem repeat domains and a C-terminal LPxTG peptidoglycan sorting signal. Serine-rich repeat glycoproteins (SRRP), like the *S. aureus* SraP protein, are another family of CWA adhesins that can mediate biofilm formations *via* intercellular adhesion (Sanchez et al., 2010; Lizcano et al., 2012). Other types of CWA proteins which have been shown to be involved in mediating biofilm formation in staphylococci include the Biofilm associated protein (Bap; Cucarella et al., 2001), the G5-E repeat family protein termed Accumulation-associated protein (Aap) in *S. epidermidis* (SasG in *S. aureus*; Rohde et al., 2005; Geoghegan et al., 2010), the *S. aureus* proteins SdrC, SasC, and Protein A (Merino et al., 2009; Schroeder et al., 2009; Barbu et al., 2014), the *S. epidermidis* protein SesC (Khodaparast et al., 2016), and the NEAT motif family protein IsdC (Missineo et al., 2014). Also non-covalently attached cell surface proteins, like the bifunctional autolysin/adhesins AtlE and Aae (Heilmann et al., 1997, 2003) and the giant (1 MDa) protein termed Extracellular matrix binding protein (Embp) in *S. epidermidis* (Ebh in *S. aureus*; Christner et al., 2010), have been shown to mediate staphylococcal biofilm formation. It has been shown that the sensitivity of biofilms to enzymes, can indirectly be used as a method to find the nature of the matrix of the biofilm (Chaignon et al., 2007; Fredheim et al., 2009).

Food associated *Staphylococcus* spp. can form both *ica*-dependent and *ica*-independent biofilms (Møretrø et al., 2003; Rode et al., 2007). It has been suggested that *ica*-independent biofilm formation of staphylococci from mastitis was connected to the presence of the gene encoding the Bap, but this mechanism seems to be less frequent in staphylococci from other sources (Cucarella et al., 2001; Vautor et al., 2008).

Benzalkonium chloride (BC), a quaternary ammonium compound (QAC), is widely used in disinfectants in the food industry and in healthcare facilities (Tezel and Pavlostathis, 2015). A number of bacteria have been reported to harbor genes encoding membrane protein eﬄux pumps that can export and provide increased tolerance to BC. In staphylococci six different eﬄux proteins (QacA, QacB, QacC, QacG, QacH, and QacJ) have been reported and shown to be widely spread in strains of both clinical and food origin (Wassenaar et al., 2015).

All Qac eﬄux proteins provide staphylococci with low-level tolerance to BC and other QACs (Furi et al., 2013; Wassenaar et al., 2015). Typical minimal inhibitory concentrations (MIC) of staphylococci expressing Qac proteins are in the range 4–12 ppm compared to MIC-values ≤2 ppm for sensitive strains (Heir et al., 1995). These tolerance levels are much lower than the lowest concentration of QAC used in the food industry, which is typically above 200 ppm (Tezel and Pavlostathis, 2015). It has been shown that staphylococci in biofilms have higher tolerance to QAC compared to planktonic phase staphylococci (Campanac et al., 2002). However, whether the presence of *qac* genes may be advantageous for staphylococci in biofilms and under food industry relevant conditions and concentrations when exposed to QAC, has to our knowledge not been reported.

In the present study, the biofilm matrix composition of *Staphylococcus* spp. isolated from the food industry was determined using enzymes targeting specific matrix components. Genetic determinants for biofilm associated and cell-wall anchored (CWA) proteins were investigated by whole genome sequencing. Furthermore the effect of the composition of the biofilm matrix as well as the presence of *qac* resistance genes on the efficacy of the disinfectant BC was studied.

## Materials and Methods

### Bacterial Strains and Growth Conditions

A collection of 35 staphylococci, from food (eight strains) or food processing environments (27 strains) from the Nofima strain collection were used in initial screening for biofilm formation. The eight strains identified as capable of forming biofilms and subjected to further characterization are listed in **Table 1** along with the reference strains used. Unless stated otherwise the bacteria were stored at -80°C and cultured at 30°C on tryptic soy agar (TSA) or TSB with shaking. For *S. aureus* RN4220/pSK265 and RN4220/*qacC*, chloramphenicol (6 ppm, final concentration) was included in the growth medium of overnight cultures used in the experiments.

**Table 1:** *Staphylococcus* strains used in this study.

Strain no.^1^ (species)	Origin	Other designations and characteristics	Reference
**Food and food industry strains**			
MF1767 (*Staphylococcus lentus*)	Poultry processing equipment		Sundheim et al., 1992
MF1862 (*S. lentus*)	Poultry		Sundheim et al., 1992
MF1844 (*S. cohnii*)	Poultry processing equipment		Sundheim et al., 1992
MF4371 (*S. saprophyticus*)	Salmon processing plant		Schirmer et al., 2012
MF6029 (*S. saprophyticus*)	Meat processing equipment	Isolate 12; *qacC*^2^	Sundheim et al., 1992; Heir et al., 1995
MF1871 (*S. capitis*)	Bakery industry product	Isolate 6; *qacA*^2^	Heir et al., 1995
MF1872 (*S. capitis*)	Poultry	Isolate 7; *qacA*^2^	Sundheim et al., 1992; Heir et al., 1995
MF1789 (*S. epidermidis*)	Poultry processing equipment		Sundheim et al., 1992
**Control strains**			
ATCC35984 (*S. epidermidis*)		RP62A	Christensen, 1982
RN4220 (*S. aureus*)			Kreiswirth et al., 1983
RN4220/pSK265 (*S. aureus*)		Strain RN4220 with plasmid cloning vector pSK265	Jones and Khan, 1986; Heir et al., 1998
RN4220/*qacC* (*S. aureus*)		Strain RN4220 with *qacC* cloned into pSK265	Heir et al., 1998

^1^*MF numbers refer to Nofima’s strain collection. ^2^Previous designation and strain characteristics according to Heir et al. (1995).*

### Biofilm Assay

Biofilm formation was assayed by cultivation in microtiter plates (Falcon) in 200 μl TSBNG [Tryptic Soy Broth (Oxoid) + 0.33 % glucose + 0.26 % NaCl; modified from Schwartz et al., 2012] at 30°C for 48 h. The suspensions were poured off and the plate was washed with dH_2_O with a plate washer (Wellwash AC, Thermo Electron Corporation). After the washing 200 μl 0.1 % crystal violet (Merck) was added and after 4 min the plates were washed again to remove non-binding crystal violet. Two hundred microliters of ethanol added 0.2% HCl (37%) was added to release crystal violet, incubated for 2 min with shaking, before 100 μl was transferred to a new microtiter plate, and OD_600__nm_ was measured (SpectroStar Nano, BMG Labtec) as an indicator for biofilm formation.

### Effect of Enzymes and Chlorine on Biofilm Detachment

Biofilms were grown in microtiter plates in TSBNG for 48 h as described above. The suspension was poured off and the plate washed with dH_2_O with a plate washer. For each strain 200 μl enzyme or chlorine solution was added to three parallel wells. The following enzymes were tested (final concentrations in parentheses). Dispersin B (50 μg/ml, Kane Biotec Inc), DNAse I (100 μg/ml, Sigma–Aldrich), Proteinase K (100 μg/ml, Sigma–Aldrich) and Trypsin (100 μg/ml, Sigma–Aldrich). Dispersin B, a glycoside hydrolase, is known to degrade polysaccharide matrix (Itoh et al., 2005), DNase I degrades eDNA (Qin et al., 2007) and Proteinase K and trypsin are able to degrade protein-based biofilm matrix (Chaignon et al., 2007). Concentrations were chosen based on previous studies (Itoh et al., 2005; Kogan et al., 2006; Chaignon et al., 2007; Harmsen et al., 2010). A solution of 0.03% chlorine, pH 12 was made by dilution from hypochlorite (Klorin, Lilleborg, Oslo, Norway) and by addition of NaOH). Alkaline chlorine based cleaning agents are among the most commonly used in the food industry (Fukuzaki, 2006). For controls, 200 μl phosphate buffered saline (PBS) were added to five parallel wells. The biofilms were exposed for 2 h at 30°C on a rolling table. The suspensions were poured off and the plates were washed and stained with crystal violet and treated as described above before measurement of the remaining biofilm as OD_600nm_. The degree of detachment was calculated by comparing enzyme treated and PBS (control) treated biofilms.

### Genome Sequencing and Assembly

DNA isolation, genome sequencing and *de novo* genome assembly was performed as previously described (Fagerlund et al., 2016), with paired-end 2 × 300 bp reads on a MiSeq instrument (Illumina). Contigs with size < 200 bp and with coverage < 15 were removed from the assemblies. The sequences were annotated using the NCBI Prokaryotic Genomes Automatic Annotation Pipeline (PGAAP) server^1^. All sequence data associated with this project have been deposited at NCBI under the BioProject ID PRJNA311173.

### Sequence Analysis

Identification at the species level was confirmed by RDP search of the 16S rRNA genes from the whole genome assemblies^2^.

The publicly available genome sequences of *S. epidermidis* ATCC 35984 (GenBank accession CP000029), the ATCC 35984 pSERP plasmid (CP000028), the complete genome sequence of *S. aureus* NCTC 8325 (CP000253), from which *S. aureus* RN4220 is derived (Herbert et al., 2010), in addition to the draft genome of *S. aureus* RN4220 (AFGU01000000), were included in the analyses. The genome sequences were downloaded from the GenBank database^3^.

The genomes were analyzed for the presence of genes of interest using BLAST+ v2.2.30 (Camacho et al., 2009). Proteins selected for use as query sequences fitted one of three criteria: (i) QAC eﬄux pump proteins, (ii) proteins known to be associated with biofilm formation in staphylococci, including proteins known to function as intercellular adhesins or (iii) surface bound proteins possessing LPxTG anchoring motifs (LPxTG). The last criterium was included since it is known that CWA proteins may promote biofilm formation in *S. aureus* and *S. epidermidis*, and the presently analyzed genomes belonging to other *Staphylococcus* spp. may potentially employ novel CWA adhesin proteins during biofilm formation. The list of proteins used as queries in BLAST search is listed in Supplementary Materials. The annotated proteins in each genome assembly were subjected to a Pfam domain search to identify proteins with YSIRK type signal peptide (PF04650) and the Gram positive anchor (PF00746) domains.

Predicted protein function was assessed using the InterProScan tool^4^. Alignments were created using CLC Main Workbench 7.5 (CLCbio). Protein structure prediction was performed using homology modeling methods based on sequence profiles generated by iterative BLAST searches, using the Phyre2 prediction server (Kelley and Sternberg, 2009).

Assembly of genome sequences from Illumina reads often results in gaps in the genome assembly at repetitive sites, like, e.g., the sequences of genes encoding large proteins with tandemly repeated domains. When loci containing partial genes next to gaps in the assembly were investigated, the initial partial genes (and subsequently identified matching sequences) were used as queries in Blastn searches against the genome assembly sequences. Obtained search hits were aligned to assess whether they were likely to represent segments of the same gene. When more than one locus next to different assembly gaps encoded identical repeat domains the loci were considered likely to belong to the same gene.

### Minimal Inhibitory Concentration of Benzalkonium Chloride

An overnight culture in TSB was diluted 1:100 in TSBNG and 20 μl was added to the wells of 100-well plates (Oy Growth Curves Ab Ltd) with 180 μl of BC (Sigma–Aldrich) diluted in TSBNG, resulting in final concentrations of BC of 1, 2, 4, 6 and 8 ppm. The plates were incubated at 30°C for 20 h and the optical density measured automatically every 10 min (with 10 s shaking before each measurement) using a Bioscreen FP-1100-C (Oy Growth Curves Ab Ltd). The MIC was calculated using a cut-off value for detectable growth of OD_600__nm_ 0.1 after 20 h.

### Lethal Effect of Benzalkonium Chloride on Biofilms

The lethal effect of user-concentrations of BC (200 ppm) was determined against biofilms grown on stainless steel. A stainless steel coupon (AISI 304 2B) of 2 cm × 2 cm was placed in each well of a six wells tissue culture plate. The well was added 5 ml overnight culture diluted in TSBNG to approximately 10^7^ cfu/ml. After an attachment phase of 3 h at 30°C, the suspension was removed and the coupons rinsed gently with sterile distilled water. The rinsed coupons were placed in new wells, 3 ml TSBNG added, and the biofilms grown at 30°C for 48 h. After incubation, the suspensions were pipetted off and the coupons rinsed gently with dH_2_O. The biofilms were exposed to 6 ml 200 ppm BC. Controls were added 6 ml dH_2_O. After 5 min exposure at room temperature the coupon was transferred to a glass tube with 6 ml Dey Engley Neutralizing broth (Difco). The tube with the coupon was sonicated (40 Hz) for 10 min to dislodge the bacteria, then 34 ml Dey Engley neutralizing broth was added and the number of cfu determined after serial dilution and plating to TSA.

### Disinfection Suspension Test of Benzalkonium Chloride

The effect of BC was tested in a modified European suspension test (CEN, 1997). An overnight culture in TSBNG was diluted 10 times with peptone water and 0.5 ml of the resulting suspension was transferred to 4.5 ml with 10 ppm benzalkonium chloride or sterile dH_2_O (control). After 5 min exposure to BC at room temperature, 0.5 ml of the suspensions were transferred to new tubes with 4.5 ml Dey/Engley Neutralizing broth. Dilution series were made in peptone water and the number of surviving bacteria determined by plating to TSA. Log reductions were calculated by comparing BC treated suspensions with controls.

### Statistical Analysis

Minitab^®^(v16.1.1, 2010^5^) was used to calculate statistical significance of differences between groups (2-sample-*t*-test). The mean values of technical replicates were calculated and statistical tests based on the variation between the biological replicates. Standard errors were calculated in Microsoft Excel.

## Results

### Detachment by Enzymes Targeting Specific Matrix Components

**FIGURE 1:**
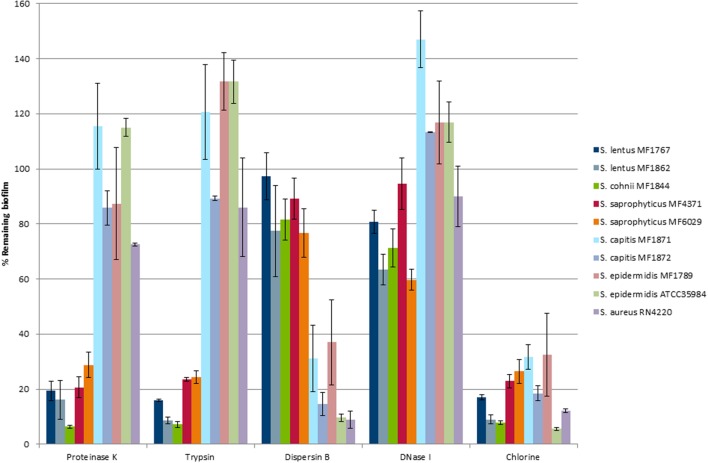
**Remaining biofilm (%) after treatment with enzymes and chlorine for ten *Staphylococcus* spp. strains.** Data presented are means and standard errors of percent remaining biofilm after treatment compared to control (PBS treatment) for triplicate experiments.

Eight strains (**Table 1**) formed biofilms (OD_600__nm_ > 0.2) out of a collection of 35 staphylococci isolated from food and food processing environments. The effect of the enzymes Dispersin B, DNase I, Proteinase K, and trypsin on the detachment of preformed biofilms was tested for these eight strains and for the reference strains *S. epidermidis* ATCC 35984 and *S. aureus* RN4220, known as strong biofilm formers harboring *ica*-genes (Ziebuhr et al., 1999; Møretrø et al., 2003; You et al., 2014) (**Figure 1**). Based on the detachment pattern after exposure to enzymes, these ten strains could be clustered into two groups. Biofilms of five strains (*S. lentus* MF1767 and MF1862, *S. cohnii* MF1844, and *S. saprophyticus* MF4371 and MF6029) were strongly disrupted upon treatment with Proteinase K and trypsin, while little effect was observed upon treatment with the glycoside hydrolase Dispersin B. For simplicity, this group was termed “protein biofilm group” based on literature showing that this phenotype is associated with strains that produce a biofilm matrix primarily consisting of proteins and not polysaccharides (Jabbouri and Sadovskaya, 2010). In contrast, biofilms made by the strains *S. capitis* MF1871 and MF1872, *S. epidermidis* MF1789 and ATCC 35984, and *S. aureus* RN4220 detached upon treatment with Dispersin B, but not upon treatment with Proteinase K or trypsin (**Figure 1**). For simplicity, these strains were termed as belonging to “PIA biofilm group.” A detachment effect (*p* = 0.014) of DNase I was observed for the strains belonging to the protein biofilm group (26% mean detachment), while no effect (*p* = 0.14) was observed for the strains in the PIA biofilm group. Chlorine had a strong detachment effect on biofilms of all strains and there were no significant differences in effect of chlorine on biofilm detachment between the two groups (**Figure 1**; *p* = 0.61).

### Genome Sequencing and Analysis

The genomes of the eight biofilm producing staphylococci (**Table 1**) were sequenced to examine the presence of specific biofilm- or matrix-associated genes and BC resistance determinants (see below). The main general features of all eight genome assemblies are shown in Supplementary Table S1. The genome sizes ranged from 2.5 to 2.7 Mb and the GC content ranged from 31.8 to 33.1%, which is in the range typically found in *Staphylococcus* spp. genomes (Suzuki et al., 2012).

Genome sequence analyses showed that all five strains of the PIA biofilm group contained the complete *icaR-icaADBC* locus required for production of PIA. The *ica* genes were not found in any strains from the protein biofilm group. Genes encoding putative additional Baps are summarized in **Table 2**, with additional information detailed in Supplementary Table S2 and further described below.

**Table 2:** Presence of genes potentially associated with biofilm formation, including cell wall anchored (CWA) proteins^a^.

	Protein biofilm group					PIA biofilm group				
Species	*S. lentus*		*S. cohnii*	*S. saprophyticus*		*S. capitis*		*S. epidermidis*		*S. aureus*
Strain	MF1767	MF1862	MF1844	MF4371	MF6029	MF1871	MF1872	MF1789	ATCC 35984	RN4220^b^
**Locus tag prefix**	**AXY34_**	**AXY37_**	**AXY36_**	**AXY40_**	**AXY41_**	**AXY38_**	**AXY39_**	**AXY35_**	**SERP**	**SAOUHSC_^b^**

PIA	–	–	–	–	–	*icaR* *icaADBC*	*icaR* *icaADBC*	*icaR* *icaADBC*	*icaR* *icaADBC*	*icaR* *icaADBC*
Embp/Ebh	–	–	–	–	–	*embp*^cd^	*embp*^d^	*embp*^d^	*embp*	*ebh*
SasC/Mrp/FmtB domain proteins	–	–	–	–	–	*sesA* *sesG*	*sesA* *sesG*^c^	*sesA*	*sesA* *sesG*	*sasB* *sasC*
Bap family	–	–	*bap*^d^	–	–	–	–	–	*bhp*	–
Serine-aspartate repeat proteins	*clfB*	*clfB*	–	–	–	*sdrX*^c^ *sdrZL*^c^	*sdrX*^c^ *sdrZL*^c^	*sdrE*^c^ *sdrF* *sdrG* *sdrH*	*sdrF*^d^ *sdrG* *sdrH*	*clfA* *clfB* *sdrC* *sdrD*
FnBPA, FnBPB	–	–	–	–	–	–	–	–	–	*fnbA* *fnbB*
*S. saprophyticus* surface protein G	–	–	–	*sssG*^c^	–	–	–	–	–	–
Uro-adherence factor A	–	–	–	*uafA*^c^	*uafA*^c^	–	–	–	–	–
*S. lentus* surface protein A	*slsA*^c^	*slsA*^c^	–	–	–	–	–	–	–	–
*S. cohnii* surface protein E	–	–	*scsE*^c^	–	–	–	–	–	–	–
*S. epidermidis* surface protein C	–	–	–	–	–	*sesC*	*sesC*	*sesC*	*sesC*	–
G5-E repeat family protein	–	–	–	–	–	–	–	–	*aap*	*sasG*
Three-helical bundle protein	–	–	–	–	–	*spa*^d^	*spa*^d^	–	–	*spa* *spi*
SRRP proteins	–	12885^c^	–	–	–	*sraP*^c^	*sraP*^c^	*sraP*	*sraP*	*sraP*
*S. saprophyticus* surface protein F	–	–	*sssF*	*sssF*	*sssF*	–	–	–	–	–
NEAT domain proteins	*isdC* 10605 12645	*isdC* 06145 05890	–	–	–	*isdC* 05010 04985 04990	*isdC* 11495 04030 04025	–	–	*isdC* *isdA* *isdB* *isdH*
Other LPxTG domain proteins	–	–	09850 09225 11360^c^	–	12070	*sesB* *sesE*-like *sesH*-like 10330	*sesB* *sesE*-like *sesH*-like 10295	*sesB* *sesE* *sesH*	*sesB* *sesE* *sesH* *sesI*	*sasD*^d^ *sasF* *sasH*
Small basic protein (Sbp)	09055	01990	08170	06645	04110	11465	05970	05425	*sbp*	00617
Bifunctional autolysin AtlE	06865^c^	08990^c^	03795^c^	*aas*	*aas*^c^	*atlE*	*atlE*	*atlE*	*atlE*	*atl*
Multifunctional autolysin Aae	00485	01440	11620	10120	09065	07140	07640	*aae*	*aae*	*aaa*
Eap/Emp	–	–	–	–	–	–	–	–	–	*eap* *emp*

^a^*For each identified gene, the gene name or locus tag is listed. The locus tag prefixes for each strain is listed below the strain names in the table header. Further details are found in Supplementary Table S2. ^b^Locus tags are obtained from the genome of S. aureus NCTC 8325 (CP000253), from which S. aureus RN4220 is derived (Herbert et al., 2010), since the publicly available genome sequence of RN4220 (AFGU01000000) is not annotated. All listed genes were also present in RN4220. ^c^The predicted protein is encoded on multiple contigs. For further details, see Supplementary Table S2. ^d^The gene encodes a truncated protein and/or contains an internal stop codon.*

A gene encoding homologs to the Small basic protein (Sbp) reported to be critical for biofilm formation in *S. epidermidis* (Decker et al., 2015), and genes encoding homologs to the two reported autolysin/adhesins AtlE and Aae were found to be conserved across all analyzed genomes.

### Putative Biofilm Associated Genes Present in the Protein Biofilm Group

**FIGURE 2:**
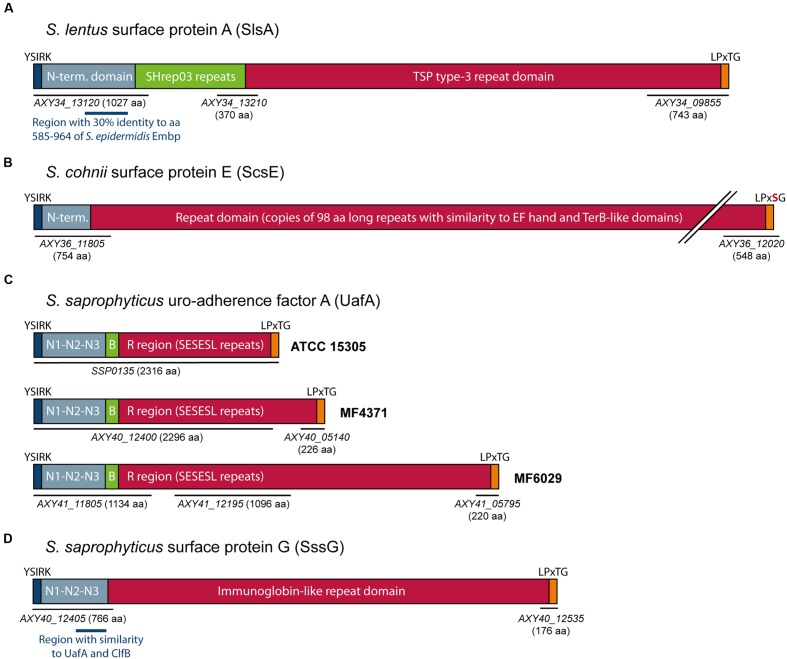
**Cell-wall anchored proteins identified in food associated *Staphylococcus* spp. strains producing proteinaceous biofilms (A–D).** The diagrams show the predicted organization of protein subdomains. All proteins except UafA from strain ATCC 15305 (in panel **C**) were encoded on multiple contigs in the genome assemblies. Selected loci encoding specific protein sections are indicated by black bars below each diagram, and selected regions of similarity with known biofilm associated proteins are shown with blue bars. The relative length of each diagram is meant to illustrate the size of the proteins estimated from the length and read coverage of the contigs encoding each protein. However, in **(B)**, for clarity, the repeat domain of ScsE is shortened relative to the estimated length.

The two *S. lentus* strains MF1767 and MF1862, which belonged to the protein biofilm group, each encoded homologs to ClfB and IsdC, known to mediate biofilm formation under specific conditions (Abraham and Jefferson, 2012; Missineo et al., 2014). In both *S. lentus* genomes, we also found evidence of a large CWA protein, encoded on several different contigs, which we will refer to as *Staphylococcus lentus* surface protein A (SlsA). (**Table 2** and **Figure 2A**). The N-terminal parts of SlsA containing YSIRK signal peptide domains were encoded by genes AXY34_13120 and AXY37_12645. These partial proteins showed about 30% identity at the amino acid level to the N-terminal domain of *S. epidermidis* Embp protein in an alignment covering ~400 amino acids (aa). They also contained tandem copies of a 90 aa long repeat sequence similar to those referred to as SHrep03 repeats in the protein encoded at locus SH1471 in *Staphylococcus haemolyticus* strains JCSC1435 (accession AP006716). In both strains, ORFs containing copies of the SHrep03 repeat and tandem TSP type 3 repeat domains (IPR028974) were encoded on short contigs (AXY34_13210 and AXY37_12875), predicted to represent the central part of *slsA*. The putative C-terminal of each protein, with tandem TSP type 3 repeat domains and a Gram positive anchor domain containing a LPxTG motif was identified next to a gap in each assembly (AXY34_09855 and AXY37_10540). In addition, several short contigs containing ORFs harboring the SHrep03 and TSP type 3 repeat domains were identified. When considering the length and assembly coverage for the identified contig fragments covering this putative gene, the length of a putative intact gene was estimated to be about 20 Kbp, which would correspond to a protein almost 7000 amino acids in length.

One of the *S. lentus* strains; MF1862, additionally contained a second partial gene, AXY37_10705, located next to an assembly gap and which encoded a protein with an LPxTG anchor motif. This protein contained repeats similar to those found in SRRPs such as SraP of *S. aureus*, which have been shown to promote biofilm formation in microtiter plates (Sanchez et al., 2010). The MF1862 genome additionally contained four short contigs encoding single ORFs harboring serine-rich repeats similar to those found in AXY37_10705. Located downstream of AXY37_10705 were two genes encoding glycosyltransferases GtfA and GtfB, which are involved in the first step of SRRP glycosylation, however, the MF1862 genome did not encode the accessory Sec proteins usually associated with SRRP genes in other species (Lizcano et al., 2012).

The third strain belonging to the protein biofilm group, *S. cohnii* MF1844 (**Figure 1**), contained several genes encoding putative CWA proteins (**Table 2**). One of these genes, AXY36_09850, encodes a 1123 aa long LPxTG protein containing four MucBP (MUCin-Binding Protein) domains (PF06458). A second locus contained two neighboring genes (AXY36_12050 and AXY36_12055) which encode protein fragments with around 60% identity toward regions 1–937 and 1467–2164, respectively, of the 2276 aa long Bap from *S. aureus* V329 (AAK38834; Cucarella et al., 2001). However, the segment aligning to *S. aureus bap* contains an internal stop codon in the region encoding the spacer fragment separating the N-terminal B region of Bap from the C repeat domain. This presumably renders the *bap* gene non-functional in MF1844.

*Staphylococcus cohnii* MF1844 also harbored sequence fragments strongly indicating the presence of a large CWA protein with a large central domain containing tandem repeats, flanked by a non-repetitive N-terminal domain and a C-terminal anchor domain (**Table 2** and **Figure 2B**). We will refer to this protein as *Staphylococcus cohnii* surface protein E (ScsE). The C-terminal of ScsE was encoded at locus AXY36_12020, located about 5 Kb upstream of the locus showing homology to *bap*, and contained a non-canonical LPxSG cell-wall sorting domain. The N-terminal domain containing an YSIRK type signal peptide sequence was encoded by the partial gene at locus AXY36_11805. These partial protein sequences have lenghts of 754 and 549 aa, respectively, and align with 99 and 97% identity toward the corresponding parts of a 3192 aa long uncharacterized protein encoded at locus XA21_08340 in *S. cohnii* strain 532 (accession LATV01000000). Two additional homologs were found in *S. cohnii* strain 57 (LATU01000000) and *S. cohnii* strain hu-01 (AYOS02000000). The central region of these proteins harbor various numbers of a tandem repeat of length 98 aa, which show similarity to the protein domains named ≪EF-hand domain pair≫ (IPR011992) and ≪TerB-like≫ (IPR029024). In *S. cohnii* MF1844, 23 additional short contigs encoding single ORFs aligning to this repeat were identified. The combined lengths of these ORFs were 4717 aa, indicating that the MF1844 homolog would have a length of at least 6000 aa. However, since the 23 short contigs on average have levels of coverage over fivefold higher than the overall average assembly coverage for the MF1844 genome, a putative functional homolog in MF1844 could potentially be significantly larger than this.

The final two strains belonging to the protein biofilm group were *S. saprophyticus* MF4371 and MF6029 (**Figure 1**). Three genes encoding CWA proteins were identified in each genome (**Table 2**). Both strains encoded the MSCRAMM adhesin named uro-adherence factor A (UafA) previously described in *S. saprophyticus* ATCC 15305 (Kuroda et al., 2005; Matsuoka et al., 2011); (**Figure 2C**). The N-terminal parts of the UafA proteins, containing the YSIRK signal peptide domain, the A-region which consists of the three subdomains N1, N2, and N3, the B-region, and the first part of the low complexity Ser-Glu-rich R region (composed of SESESL-like repeats) were encoded next to assembly gaps on loci AXY40_12400 and AXY41_11805 in the genomes of MF4371 and MF6029, respectively. These ORFs showed 99% amino acid sequence identity toward UafA of *S. saprophyticus* ATCC 15305. The C-terminal regions were encoded at loci AXY40_05140 and AXY41_05795, and contained the last part of the R region and the wall-membrane-spanning regions containing LPxTG motifs, which was identical in the three strains MF4371, MF6029 and ATCC 15305. In MF4371, one additional short contig encoding the R region SESESL-like repeats was identified (AXY40_12580), while in MF6029, six such contigs were identified. The assembly coverage for these short contigs were significantly higher than the average assembly coverage for the MF4371 and MF6029 genomes, indicating that the R region of UafA in these strains were expanded compared to in UafA from ATCC 15305, in particular in MF6029 (**Figure 2C**).

*Staphylococcus saprophyticus* MF4371 appears to also encode a second MSCRAMM protein, which we will refer to as *Staphylococcus saprophyticus* surface protein G (SssG; **Figure 2D**). Fragments of the *sssG* gene were identified on four different contigs in the genome assembly. The N-terminal region of SssG (AXY40_12405) contained two adhesion domains (IPR008966) similar to those found in the N-terminal A domains of MSCRAMM proteins such as UafA, FnBPA, and ClfA. While alignments show only around 20–24% amino acid sequence identity between SssG and these MSCRAMMs, analysis using protein structure prediction methods indicates that this region of SssG adopts a fold similar to that of the ligand-binding N2-N3 domains of MSCRAMM proteins such as ClfA (PDB: 1N67), Bbp (PDB: 5CF3), and UafA (PDB: 3IRP). AXY40_12405 also contains part of the central repeat domain of SssG. Sections of the central repeat domain were also present in the locus encoding the C-terminal fragment harboring the LPxTG motif (AXY40_12535), and on two additional short contigs (AXY40_12590, AXY40_12620). Alignments of fragments encoding the central repeat domain revealed a 89 aa long repeat unit which was 62% identical and 78% similar to the immunoglobin (Ig)-like B repeats found in the central region of the *S. epidermidis* Bap family protein Bhp (Tormo et al., 2005). The two short contigs had read coverage about 20x higher than the average MF4371 assembly coverage, suggesting that SssG harbors multiple, highly identical tandemly repeated Ig-like domains. A transposase gene was located downstream of the locus encoding the C-terminal of SssG, suggesting that *sssG* is found on a mobile genetic element.

### Repertoire of Surface Proteins in the Strains of the PIA Biofilm Group

The PIA biofilm group is composed of two *S. capitis* strains and two *S. epidermidis* strains, in addition to the reference strain *S. aureus* R4220. All five strains are members of the Epidermidis–Aureus species group, and thus relatively closely related compared with the strains in the protein-biofilm group (Lamers et al., 2012). The close relationship between these strains was reflected in a similar content of cell-wall associated proteins encoded in their genomes (**Table 2**). The close relationship was particularly evident for the two *S. capitis* strains, for which the majority of analyzed proteins showed 100% identity between the two strains.

Overall, we identified 10–19 CWA proteins encoded in the genomes of the PIA-biofilm strains, which is a significantly higher number than that found in the strains of the protein-biofilm group (3–6 CWA proteins). Homologs to several CWA proteins which have previously been shown to be involved in mediating biofilm formation in microtiter plate assays, namely Aap/SasG, SdrC, SasC, SesC, and SraP, were encoded in more than one of the strains in the PIA biofilm group (Rohde et al., 2005; Geoghegan et al., 2010; Sanchez et al., 2010; Barbu et al., 2014; Khodaparast et al., 2016). Furthermore, all strains harbored homologs to genes encoding the giant protein Ebh/Embp (Christner et al., 2010). However, in the three food-associated strains in this group, the *ebh*/*embp* genes contained multiple internal stop codons separating the gene into several open reading frames, similar to what has previously been observed for *S. aureus* N325 and Mu50 (Clarke et al., 2002).

### Presence of QAC-Tolerance Associated Genes

The *S. capitis* strains MF1871 and MF1872 were previously known to contain the *qacA* gene encoding the QacA MFS multidrug eﬄux pump known to increase tolerance to multiple substrates including the biocides QAC and chlorhexidine (Heir et al., 1995). The presence of genes encoding QacA and the QacR transcriptional repressor was confirmed by WGS of both strains. The *qacR*-*qacA* genes (locus tags AXY38_11220/AXY38_11225 and AXY39_11475/AXY39_11470) were present on contigs which showed sequence similarity toward several *Staphylococcus* spp. plasmids, suggesting that the *qacA* genes in MF1871 and MF1872 were plasmid-borne. None of the other analyzed genomes contained genes encoding QacA or the highly similar QacB proteins (Wassenaar et al., 2015).

Sequence analysis furthermore showed that three of the analyzed strains contained genes encoding QacC/Smr family SMR multidrug eﬄux pumps. The two *S. saprophyticus* strains MF4371 and MF6029 encode QacJ (AXY40_12555) and QacC (AXY41_12200), respectively. Their respective *qac* genes were found on short (~3 Kbp) contigs, having higher read coverage than the average read depth for the whole genome assemblies, and which contained genes encoding a plasmid replication protein. This suggests that the *qacJ* and *qacC* genes in MF4371 and MF6029 reside on small, multicopy plasmids. In contrast, *S. cohnii* MF1844 encodes a protein (AXY36_07250) with 94% identity toward QacH (WP_019467894) which appeared to be chromosomally encoded. The *qacH* gene was present on a 404 Kbp long contig with read coverage similar to the average assembly coverage for MF1844 and which encoded typical chromosomal genes.

### Tolerance to Benzalkonium Chloride

The two food-associated *S. saprophyticus* isolates MF4371 and MF6029 harboring plasmid-encoded *qacJ* and *qacC* genes, respectively, as well *S. aureus* RN4220/*qacC* (control strain expressing *qacC*; **Table 1**), had MICs of 6–8 ppm BC. The two *qacA*-positive *S. capitis* isolates MF1871 and MF1872 and the *qac*-negative isolate *S. lentus* MF1862 had MICs of 4 ppm. The remaining isolates, including the *qacH*-positive *S. cohnii* MF1844, had MICs below 4 ppm BC.

**FIGURE 3:**
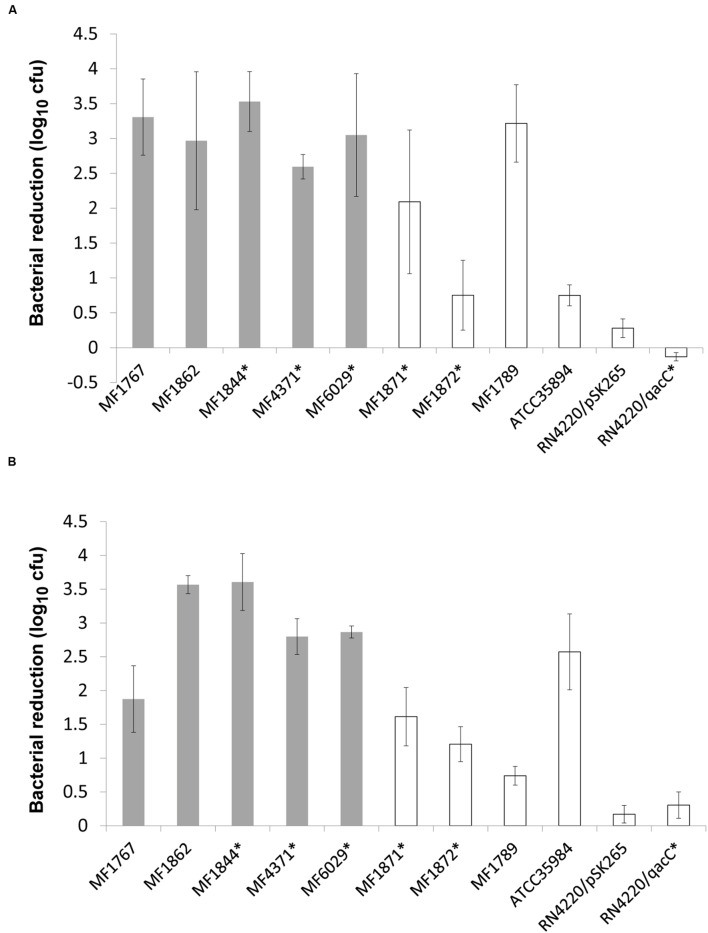
**Effect of benzalkonium chloride (BC) against *Staphylococcus* spp. strains in (A) biofilm (200 ppm BC) and (B) suspension (10 ppm BC).** Strains belonging to “protein biofilm group” in gray and “PIA biofilm group” in white. Presence of *qac* genes is indicated with asterisk after strain name. Means and standard errors are shown.

The strains that formed protein-dependent biofilms (biofilms degraded by proteinase) were more susceptible to the lethal action of BC in biocidal tests than strains producing biofilms degraded by the glycoside hydrolase Dispersin B. This difference was significant both in biofilms (*p* = 0.04; **Figure 3A**) and in suspensions (*p* = 0.014; **Figure 3B**). There were no differences (*p* = 0.89 for biofilm, *p* = 0.73 for suspension) in susceptibility toward BC between strain containing *qac* genes and the other strains. The strains were more susceptible to BC in suspension than in biofilm.

## Discussion

### Biofilm Formation in Food Associated Staphylococci

As has also been shown in other studies, the frequency of food associated staphylococci showing strong biofilm formation *in vitro* was low compared to what has been reported for clinical/human strains, even when using methodology that is optimized with high salt and sugar concentrations and temperatures allowing growth (Møretrø et al., 2003; Jaglic et al., 2010). Also, in a survey of attached microbiota from dairies, it was concluded that since only one out of eight staphylococci isolated were strong biofilm formers, biofilm formation was unlikely an explanation for survival on milk contact surfaces (Cherif-Antar et al., 2016). In the present study, five out of nine poultry associated CNS belonging to four different species were strong biofilm formers, suggesting an association between biofilm formation abilities and poultry origin. However, a larger collection of strains would be necessary to confirm this.

### Resistance toward BC

*Staphylococcus*, especially coagulase-negative species are among the most frequently isolated bacteria from food processing surfaces and survival after both cleaning and disinfection has been explained by specific resistance mechanisms and formation of a protective biofilm matrix (Langsrud, 2009). As also shown by others (Campanac et al., 2002; Luppens et al., 2002) biofilm formation can protect cells from disinfection, illustrated by a similar range of bactericidal effect at 10 ppm BC in suspension tests and 200 ppm (user-concentration) in biofilm tests. One explanation for the observed protection is that the bactericidal agent does not reach the target cells because of reduced diffusion and/or neutralization of the compounds by the matrix (Bridier et al., 2011). Interestingly, not only biofilm in itself, but the matrix composition appeared to affect bacterial resistance as strains belonging to the protein biofilm group were generally more susceptible than those belonging to the PIA biofilm group. This suggests that a biofilm matrix dominated by polysaccharides protects staphylococci against BC better than a matrix dominated by proteins. One possible explanation is reduced diffusion of the positively charged BC in a biofilm in which the negatively charged PIA is a major matrix component, a resistance mechanism that has been suggested also by others (Ganeshnarayan et al., 2009). It should be pointed out that the difference in BC susceptibility between the two groups were not restricted to biofilms, but also appeared in suspension. This indicated that other, intrinsic mechanisms could be involved, or that PIA to a certain extent is also produced in suspension (Vandecasteele et al., 2003). Also, one of the PIA biofilm strains showed an equal level of sensitivity to BC as the protein biofilm group strains. Together, the large variances in phenotypic resistance patterns observed reflected the profound genomic differences between strains (see below).

Differences in tolerance to BC in staphylococci have traditionally been explained by the presence of *qac* genes encoding eﬄux pumps. Apparently, biofilm growth is a much more powerful resistance mechanism than these eﬄux mechanisms. In accordance with recent results obtained by Furi et al. (2013), we observed no protective effect of *qac*-genes in bactericidal tests against BC in biofilms or in suspension. Nevertheless, our results supported earlier reports about the role of *qac* genes for the ability to grow in the presence of low concentrations of BC (Furi et al., 2013; Skovgaard et al., 2013; Marchi et al., 2015). *S. cohnii* MF1844 was susceptible to BC, despite harboring a *qacH*-like gene. This could be due to a lower gene copy number, low gene expression or less effective eﬄux mechanism compared to similar pumps. The intermediate susceptibility of the *qac*-negative *S. lentus* may be due to unknown eﬄux mechanisms or resistance acquired, e.g., from adaptation. The biofilms of all strains were equally removed by user-concentrations of alkaline chlorine, which is a frequently used cleaning agent in the food industry. Chlorine has broad activity, can dissolve and remove proteins, polysaccharide, DNA, and lipids (Fukuzaki, 2006), and has been shown to eradicate biofilms of MRSA (Lee et al., 2009). Whether the hypochlorite treatment can level out differences in susceptibility to disinfectants should be further studied.

### Strains Showing PIA-Dependent Biofilm Formation

Phylogenetically, the species of the genus *Staphylococcus* may be divided into 15 cluster groups and six species groups (Lamers et al., 2012). The four CNS strains in the PIA biofilm group were all members of the Epidermidis cluster group, belonging to the Epidermidis–Aureus species group. The *ica* locus has been found in several different staphylococcal species (Cramton et al., 1999; Møretrø et al., 2003) but its presence does not necessarily lead to PIA production since expression is regulated in response to environmental conditions (Arciola et al., 2015). In the current study, the growth medium was supplemented with glucose and sodium chloride to promote PIA production (Ammendolia et al., 1999; Rode et al., 2007) and all *ica*-positive biofilm forming strains produced a biofilm matrix that was detached by Dispersin B (**Figure 1**). This suggested that PIA was a main structural component of the biofilm matrix in these strains. For the *ica*-positive control strain *S. epidermidis* ATCC 35984, this result was in accordance with previous findings (Chaignon et al., 2007).

Homologs to a number of genes encoding proteins that have been associated with staphylococcal biofilm formation under conditions similar to those used in the current study, including *aap*/s*asG*, *sdrC*, *sasC*, *sesC*, *spa*, *sraP*, and *embp* (**Table 2**) were found in strains belonging to the PIA biofilm group. However, since the biofilms formed by this group of strains were almost completely eradicated upon treatment with Dispersin B (**Figure 1**), these proteins did not appear to be able to compensate for the loss of structural stability seen upon degradation of PIA in the biofilm matrix. Further examination would be required to determine whether any of these proteins nevertheless does contribute to one or more of the stages during biofilm development in these strains.

### CNS Strains Producing Proteinaceous Biofilm Matrix

Due to their relevance as human pathogens, biofilm formation has been extensively investigated in *S. epidermidis* and *S. aureus* (Arciola et al., 2015), while in contrast, much less is presently known about the mechanisms of biofilm formation in more distantly related CNS strains. However, proteinaceous biofilms have earlier been reported for several CNS strains outside of the Epidermidis cluster group, including *S. lugdunensis*, *S. haemolyticus*, and *S. cohnii* (Chaignon et al., 2007; Fredheim et al., 2009; Potter et al., 2009). The five CNS strains from the current study producing *ica*-independent biofilms were identified as *S. cohnii* and *S. saprophyticus*, belonging to the Saprophyticus species group, and *S. lentus*, which belongs to the Sciuri species group (Lamers et al., 2012). With the exception of homologs to the three biofilm associated genes encoding Sbp and the autolysin/adhesins AtlE and Aae, present in all ten examined strains (regardless of their sensitivity to Dispersin B and proteinases), and the genes encoding ClfB and IsdC, found in the two examined *S. lentus* strains, no genes encoding known Baps were identified in the genomes of the strains in the protein biofilm group in the current study (**Table 2**). The presence of *sbp* and *atlE*/*aae* is probably required, but not sufficient, for biofilm formation. Furthermore, ClfB and IsdC only appears to mediate biofilm formation in the absence of calcium and under low-iron growth conditions, respectively (Abraham and Jefferson, 2012; Missineo et al., 2014), which are conditions not encountered in the current study. It therefore seems likely that yet undescribed mechanisms may account for the observed ability of these strains to build a biofilm.

### Search for Putative Novel Biofilm Associated Proteins

In *S. aureus* and *S. epidermidis*, proteins able to mediate biofilm formation in the absence of PIA are generally found to be large CWA proteins. Of these, several MSCRAMM proteins appear to play dual roles, able to act both as adhesins binding to human ECM proteins and as mediators of biofilm formation on abiotic surfaces by promoting bacterial intercellular interactions (Abraham and Jefferson, 2012; Geoghegan et al., 2013; Barbu et al., 2014). In order to identify potential proteins involved in biofilm formation in the *ica*-negative isolates examined in the current study, the genomes were screened for the presence of proteins with cell wall anchor domains, in addition to searching for homologs to genes encoding known Baps. Overall, we identified a much lower number of CWA proteins encoded in the genomes of the five *S. lentus*, *S. cohnii* and *S. saprophyticus* strains (3–6 proteins), compared with the numbers found in the five examined strains belonging to the Epidermidis–Aureus species group (10–19 proteins; **Table 2**). It should be noted that the method of WGS employed in the current study, in which *de novo* genome assemblies were generated from relatively short-read sequencing data, is known to result in gaps in the genome assembly at sites of sequence repeats. Therefore we were not surprised to find that most of the genes encoding the highly repeat-rich proteins identified in the current study were encoded on more than one contig in the genome assembly.

Both *S. lentus* strains encoded what appeared to be a large CWA protein with a C-terminal LPxTG motif, which we have referred to as SlsA (**Figure 2A**). The N-terminal domain of SlsA is similar in sequence to that of *S. epidermidis* Embp, and the central and C-terminal domains of SlsA harbor two types of repeat sequences: SHrep03 repeats and TSP type 3 repeats. To our knowledge, a protein with this domain organization has not been previously described. However, the modular domain structure composed of an N-terminal non-repetitive region followed by various repeat domains is similar to that found in several staphylococcal biofilm-associated CWA proteins. Therefore, we consider SlsA as a candidate for a specific protein responsible for the observed biofilm phenotype in the examined *S. lentus* strains. One of the *S. lentus* strains, MF1862, additionally encoded a SRRP. This protein could possibly contribute to protein-dependent biofilm formation in this strain as SRRPs are known to mediate adhesion, bacterial aggregation, and biofilm formation (Lizcano et al., 2012).

Five CWA proteins were identified in *S. cohnii* MF1844 (**Table 2**). Of these, the protein encoded at locus AXY36_09850 contained four MucBP domains, and may potentially be involved in primary attachment. Proteins containing MucBP domains have been suggested to play a role during intestinal adhesion in *Lactobacillus* spp. (Kleerebezem et al., 2010), and contribute to biofilm formation in *Streptococcus thermophilus*, (Couvigny et al., 2015). Also, a gene encoding what appears to be a very large CWA protein, which we have named ScsE (**Figure 2B**) was identified as a candidate for a novel protein capable of mediating protein-dependent biofilm formation in *S. cohnii* strains. The predicted protein contained a ~550 aa long non-repetitive N-terminal region, and multiple copies of a 98 aa long repeat showing similarity to EF-hand domain pair and TerB-like domains. Homologs to *scsE* from MF1844, encoding proteins with variable numbers of repeat domains, were found in three publicly available *S. cohnii* genome sequences. Neither the N-terminal domains nor the repeat domains from these proteins show any homology to any domains found in characterized CWA proteins known to be involved in adhesion or biofilm formation. However, as for the *S. lentus* SlsA protein, their overall domain organization is similar to that found in many staphylococcal MSCRAMM adhesins and known biofilm-associated CWA proteins. ScsE is therefore a candidate for a novel protein capable of contributing to protein-dependent biofilm formation in *S. cohnii* strains.

*Staphylococcus saprophyticus*, being a frequent cause of urinary tract infections in humans, has a repertoire of cell wall associated proteins which is slightly better described in the literature compared with that of the generally non-pathogenic *S. lentus* and *S. cohnii* (Becker et al., 2014). The CWA proteins UafA, UafB, and SdrI have been associated with adhesion in this species (Kuroda et al., 2005; Sakinc et al., 2006; King et al., 2011). Of these, only UafA was encoded in the genomes of *S. saprophyticus* strains MF4371 and MF6029. UafA is an hemagglutinin and an adhesin associated with adherence to uroepithelial cells (Kuroda et al., 2005) and has a domain structure typical of MSCRAMM adhesins, with a characteristic A region composed of subdomains N1, N2, and N3, a B region and a C-terminal Ser-Glu rich region of low complexity (**Figure 2C**) (Kuroda et al., 2005; Matsuoka et al., 2011). To our knowledge, the ability of UafA to mediate PIA-independent biofilm formation on abiotic surfaces has not been assessed. One report does, however, indicate an association between increased expression of UafA and increased biofilm formation in a microtiter plate based assay (Goneau et al., 2015), potentially suggesting that UafA may be a member of the growing list of MSCRAMMs that have been shown to be able to promote biofilm formation on abiotic surfaces through mediating intercellular adhesion. It has been suggested that the C-terminal Ser-Glu rich region of UafA may act as a stalk to present the ligand-binding A and B regions away from the bacterial cell surface (Matsuoka et al., 2011). If so, it is possible that elongated Ser-Glu rich region in the UafA homologs of MF4371 and MF6029 (**Figure 2C**) may enhance the accessibility of UafA for adhesion in these strains.

*Staphylococcus saprophyticus* MF4371 also encodes a previously undescribed CWA protein which we have referred to as SssG (**Figure 2D**). SssG has a highly interesting domain structure, containing what appears to be a N-terminal A-domain typical of those found in MSCRAMM family proteins, fused to a central domain composed of tandem repeats highly similar to those of the B-repeat region of the Bap family protein Bhp (Tormo et al., 2005). Potentially, both UafA and SssG may contribute to proteinaceous biofilm formation in strain MF4371.

Further work will be required to reveal whether any of the identified proteins described above, including SlsA, ScsE, UafA, and SssG, represent novel mechanisms of protein-mediated biofilm in CNS strains.

### DNase I Treatment Had Limited Effect on Biofilm Detachment

DNase I had a slightly adverse effect on biofilm formation for four of the five *ica*-negative strains, but no effect on the *ica*-positive strains. The reason for this difference is not clear. Possibly, eDNA is more important for the structure of the protein dominated matrices, maybe by binding to proteins and stabilizing the matrix, while the PIA dominated matrix could be more stable in absence of eDNA. PIA-dependent biofilms have a more pronounced mechanical robustness compared to protein-dependent biofilms and are significantly more stable against washing procedures (Büttner et al., 2015). For *S. epidermidis* it has been shown that eDNA is especially important in the early phases of biofilm formation (Qin et al., 2007), and this may explain why DNase I only had limited detachment effect on the mature biofilms in the present study.

## Conclusion

In the present study differences in composition of biofilm matrix of food associated staphylococci was found, and strains with a protein biofilm were more susceptible to the disinfectant BC than strains with a PIA biofilm. Several putative novel mediators of proteinaceous biofilm formation in CNS strains were identified. Genes encoding staphylococcal QAC eﬄux proteins provide increased MIC-values to BC, but their presence was not associated with increased tolerance of staphylococci to biocidal concentrations.

## Author Contributions

AF, SL, EH, MM, and TM planned and designed experiments, interpreted and discussed results. AF, SL, EH, and TM wrote manuscript. AF performed the genome analyses. MM performed biofilm studies and disinfection experiments.

## Conflict of Interest Statement

The authors declare that the research was conducted in the absence of any commercial or financial relationships that could be construed as a potential conflict of interest.

## Footnotes

1 http://www.ncbi.nlm.nih.gov/genome/annotation_prok/

2 https://rdp.cme.msu.edu/seqmatch/

3 http://www.ncbi.nlm.nih.gov/

4 http://www.ebi.ac.uk/interpro

5 http://www.minitab.com
