# Supplementary material for: Biofilm Matrix Composition Affects the Susceptibility of Food Associated Staphylococci to Cleaning and Disinfection Agents
Source: Front Microbiol. 2016 Jun 6;7:856. doi: 10.3389/fmicb.2016.00856 (PMC4893552; doi:10.3389/fmicb.2016.00856)
Supplement: Supplementary file 1 [file Presentation_1.PDF]

Supplementary Material

Biofilm matrix composition affects the susceptibility of food associated staphylococci to cleaning and disinfection agents

A. Fagerlund, S. Langsrud, E. Heir, M. I. Mikkelsen, T. Møretrø*

*** Correspondence:** Corresponding Author: trond.moretro@nofima.no

1 Supplementary Materials and Methods

1.1 Additional information about BLAST analysis

The following list of protein sequences were used as queries in BLAST analysis to search for genes of interest in the *Staphylococcus* spp. genomes. Accession numbers for each protein is given in parenthesis after the protein name.

1.1.1 QAC efflux pump proteins:

QacA (ADK23699), QacR (ADK23698), and QacC (NP_647561) (Wassenaar et al., 2015).

1.1.2 Biofilm-associated proteins, adhesins, or known LPxTG proteins:

Aae (AAW53528), Aap (AAW53239), AtlE (O33635), Bap (AAK38834), Bbp (Q14U76), Bhp (AAK29746), ClfA (Q53653), ClfB (O86476), Cna (Q53654), Ebh (Q2FYJ6), Embp (Q5HPA2), FnBPA (P14738), FnBPB (BAB43593), IcaA (AAW53175), IcaB (AAW53184), IcaC (AAW53185), IcaD (AAW53183), IcaR (AAW53174), IsdA (A6QG31), IsdB (A6QG30), IsdC (A6QG32), IsdH (Q2FXJ2), Pls (P80544), SasB (AAW38458), SasC (CAR31512), SasD (WP_020976847), SasF WP_023180220), SasG (Q2G2B2), SasK (CDP40862), SasX (CBI50053), SdrC (O86487), SdrD O86488), SdrE (O86489), SdrF (Q9KI14), SdrG (Q9KI13), SdrH (WP_002457917), SdrI (Q8KWM1), SdrX (AKL92924), SdrZL (AKL92324), SesA (AAW54644), SesB (AAW53084), SesC (NP_765787), SesE (AAW54085), SesG (AAW54798), SesH (AAW54808), SesI (AAW54982), Spa (P02976), SraP (Q2FUW1), SssF (YP_005083632), UafA (Q4A0V8), and UafB (YP_004400635) (Clarke and Foster, 2006;Fey and Olson, 2010;King et al., 2012;Speziale et al., 2014;Cameron et al., 2015).Supplementary Material should be uploaded separately on submission. Please include any supplementary data, figures and/or tables.

1.1.3 References

Cameron, D.R., Jiang, J.-H., Hassan, K.A., Elbourne, L.D.H., Tuck, K.L., Paulsen, I.T., *et al*. (2015). Insights on virulence from the complete genome of *Staphylococcus capitis*. *Front. Microbiol.* 6. doi: 10.3389/fmicb.2075.00980.

Clarke, S.R., and Foster, S.J. (2006). "Surface adhesins of *Staphylococcus aureus*," in *Advances in Microbial Physiology, Vol 51,* ed. R.K. Poole (Elsevier), 187-225.

Fey, P.D., and Olson, M.E. (2010). Current concepts in biofilm formation of *Staphylococcus epidermidis*. *Future Microbiol.* 5**,** 917-933. doi: 10.2217/fmb.10.56.

King, N.P., Sakinc, T., Ben Zakour, N.L., Totsika, M., Heras, B., Simerska, P., *et al.* (2012). Characterisation of a cell wall-anchored protein of *Staphylococcus saprophyticus* associated with linoleic acid resistance. *BMC Microbiol.* 12. doi: 10.1186/1471-2180-12-8.

Speziale, P., Pietrocola, G., Foster, T.J., and Geoghegan, J.A. (2014). Protein-based biofilm matrices in Staphylococci. *Front. Cell. Infect. Microbiol.* 4. doi: 10.3389/fcimb.2014.00171.

Wassenaar, T.M., Ussery, D., Nielsen, L.N., and Ingmer, H. (2015). Review and phylogenetic analysis of *qac* genes that reduce susceptibility to quaternary ammonium compounds in *Staphylococcus* species. *Eur. J. Microbiol. Immunol. (Bp)* 5**,** 44-61. doi: 10.1556/eujmi-d-14-00038.

2 Supplementary Tables

2.1 Supplementary Table 1: Assembly quality metrics for the *Staphylococcus* spp. genome assemblies

Species	*S. lentus*		*S. cohnii*	*S. saprophyticus*		*S. capitis*		*S. epidermidis*
Strain	**MF1767**	**MF1862**	**MF1844**	**MF4371**	**MF6029**	**MF1871**	**MF1872**	**MF1789**
No. of paired-end sequences	782391	488507	755969	768191	803737	687161	719939	1090924
Assembly size (bp)	2697194	2644425	2584187	2579487	2513242	2463311	2471539	2484601
N50	75035	76277	564298	252476	458474	157186	277807	125785
Assembled coverage (average)	57×	45×	78×	53×	69×	70×	71×	72×
Contig number [a]	86	98	75	38	35	46	45	76
GC content	31.75%	31.85%	32.49%	32.98%	33.05%	32.82%	32.80%	31.95%
No. of Genes	2657	2609	2485	2524	2456	2406	2415	2346
No. of CDS	2600	2552	2421	2461	2394	2339	2353	2284
No. of pseudogenes	45	68	67	96	86	48	51	52
No. of rRNAs (5S, 16S, 23S)	6, 1, 1	2, 1, 1	2, 2, 1	2, 1, 1	2, 1, 1	2, 1, 3	0, 2, 3	2, 1, 1
No. of tRNAs	45	49	55	55	54	57	53	54

[a] Only contigs with size >200 bp and with coverage >15 were included.

2.2 Supplementary Table 2: Presence of genes potentially associated with biofilm formation, including cell wall anchored (CWA) proteins[a]

	Protein biofilm group					PIA biofilm group				
Species:	*S. lentus*		*S. cohnii*	*S. saprophyticus*		*S. capitis*		*S. epidermidis*		*S. aureus*
Strain:	**MF1767**	**MF1862**	**MF1844**	**MF4371**	**MF6029**	**MF1871**	**MF1872**	**MF1789**	**ATCC 35984**	**RN4220**[b]
Locus tag prefix:	AXY34_	AXY37_	AXY36_	AXY40_	AXY41_	AXY38_	AXY39_	AXY35_	SERP	SAOUHSC_[b]
**PROTEIN FUNCTION**										
Polysaccharide intercellular adhesion (PIA)	-	-	-	-	-	*icaR-icaADBC* 09840-09820	*icaR-icaADBC* 08750-08730	*icaR-icaADBC* 01570-01590	*icaR-icaADBC* 2292-2296	*icaR-icaADBC* 03001-03005
ECM-binding protein homologue Ebh<br>Extracellular matrix binding protein Embp	-	-	-	-	-	*embp*[c] (58%) (5 orfs)	*embp* 00665 and 00660 (58%) (5 orfs)	*embp* 00865 (97%) (4 orfs)	***embp*** 1011	*ebh* 01447 (33%)
CWA proteins with SasC/Mrp/FmtB intercellular aggregation domain (IPR026359)	-	-	-	-	-	*sesA* 03095 (53%)<br>*sesG* 09575 (51%)	*sesA* 09270 (53%)<br>*sesG*** (51%)	*sesA* 02650 (98%)	***sesA*** 1316<br>***sesG*** 1482	*sasB* 02404<br>*sasC* 01873
Biofilm-associated protein Bap /<br>Bap homologue protein Bhp	-	-	*bap* 12050 (truncated)	-	-	-	-	-	*bhp* 2392	-
MSCRAMM family of CWA proteins:<br>Serine-aspartate repeat proteins	*clfB* 13045 (31%)	*clfB* 11085 (31%)	-	-	-	*sdrX*[c]<br>*sdrZL*[c]	*sdrX*[c]<br>*sdrZL*[c]	*sdrE*[c]<br>*sdrF* 08615<br>*sdrG* 08275 (85%)<br>*sdrH* 09290 (93%)	*sdrF* 0026 (truncated)<br>***sdrG*** 0207<br>***sdrH*** 1487	*clfA* 00812<br>***clfB*** 02963<br>*sdrC* 00544<br>*sdrD* 00545
MSCRAMM family of CWA proteins:<br>Fibronectin binding proteins FnBPA, FnBPB	-	-	-	-	-	-	-	-	-	*fnbA* 02803<br>*fnbB* 02802
MSCRAMM family of CWA proteins:<br>*S. saprophyticus* surface protein G (SssG)	-	-	-	*sssH*[c]	-	-	-	-	-	-
MSCRAMM family of CWA proteins:<br>Uro-adherence factor A (UafA)	-	-	-	*uafA*[c]	*uafA*[c]	-	-	-	-	-
CWA protein with TSP type 3 domain:<br>*S. lentus* surface protein A (SlsA)	*slsA*[c]	*slsA*[c]	-	-	-	-	-	-	-	-
CWA protein with repeat domain:<br>*S. cohnii* surface protein E (ScsE)	-	-	*scsE*[c]	-	-	-	-	-	-	-
CWA protein: *S. epidermidis* surface protein C (SesC)	-	-	-	-	-	*sesC* (10420; 45%)	*sesC* 10385 (45%)	*sesC* 10105 (98%)	***sesC*** 2264	-
G5-E repeat family of CWA proteins	-	-	-	-	-	-	-	-	***aap 2398***	sasG 02798
Three-helical bundle family of<br>CWA proteins: Protein A (Spa), Spi	-	-	-	-	-	*spa 10570 (frameshift)*	*spa 11950 (frameshift)*	-	**-**	spa 00069<br>spi 02706
SRRP proteins, close to secY2<br>and/or gtfA-gtfB genes: SraP	-	12885c	-	-	-	*sraPc (57%)*	*sraPc (57%)*	*sraP 01520 (80%)*	***sraP 2281***	sraP 02990 (48%)
CWA protein S. saprophyticus<br>surface protein F (SssF)	-	-	sssF 09840 (53%)	sssF 12000	sssF 11985 (100%)	-	-	-	**-**	-
NEAT motif family of proteins:<br>Iron-regulated surface (Isd) proteins	isdC 10600 (41%)<br>10605 (4x NEAT)<br>12645 (3x NEAT)	isdC 06140 (40%)<br>06145 (4x NEAT)<br>05890 (3x NEAT)	-	-	-	*isdC 05005 (50%)<br>05010 (1x NEAT)<br>04985 (3x NEAT)<br>04990 (1x NEAT)*	*isdC 11490 (50%)<br>11495 (1x NEAT)<br>04030 (3x NEAT)<br>04025 (1x NEAT)*	-	**-**	isdC 01082<br>isdA 01081<br>isdB 01079<br>isdH 01843
Other CWA proteins with<br>LPxTG anchor domain	-	-	09850 (MucBP)<br>09225<br>11360**		12070 (peptidase)	*sesB (08175, 56%)<br>05000c (sesE-like; 37%)<br>ssp5c (sesH-like; 35%)<br>10330 (115 aa)*	*sesB 10685 (56%)<br>11485c (sesE-like; 37%)<br>ssp5c (sesH-like; 37%)<br>10295 (115aa) (100%)*	*sesB 10015 (99%)<br>sesE 03445 (89%)<br>sesH 08615 (86%)*	***sesB 2162<br>sesE 0719<br>sesH 1483<br>sesI 1654***	sasD 00094<br>(truncated)<br>sasF 02982<br>sasH 00025
Small basic protein (Sbp)	09055 (32.4%)	01990 (32.4%)	08170 (60.4%)	06645 (56.5%)	04110 (56.5%)	*11465 (78.1%)*	*05970 (78.1%)*	*05425 (98.8%)*	***sbp 0270***	00617 (60%)
Bifunctional autolysin Atl, AtlE, Aas	06865c (~34%)	08990c (~34%)	03795c (~47%)	aas 01955 (44%)	aasc (44%)	*atlE 04595 (65%)*	*atlE 04420 (65%)*	*atlE 05740 (98%)*	***atlE 0636***	atl 00994 (60%)
Multifunctional autolysin Aaa, Aae	00485 (63%)	01440 (63%)	11620 (72%)	10120 (70%)	09065 (70%)	*07140 (84%)*	*07640 (84%)*	*aae 07230 (100%)*	***aae 0100***	aaa 00427 (76%)
Secreted non-covalently attached proteins<br>relevant for biofilm formation: Eap and Emp	-	-	-	-	-	-	-	-	**-**	eap 02161<br>emp 00816

[a] For each of the identified genes, the gene name and/or locus tag is indicated. The locus tag prefixes for each strain is listed below the strain names in the table header. In the cases where a gene is predicted to span several contigs in the assembly, only the gene name or locus tag for the N-terminal fragment is listed in the table (additional locus tags are listed in the footnote). For selected loci, the amino acid sequence identity is given in parenthesis after gene name/locus tag. In these cases, the identity value is relative to the protein encoded by the orthologous gene listed in red bold in the same row, and obtained using global alignments. Additional information listed in parenthesis after the gene name/locus tag for selected genes include information on domains present in the encoded protein and information on internal stop codons and frameshifts.

[b] Locus tags are obtained from the genome of *S. aureus* NCTC 8325 (CP000253), from which *S. aureus* RN4220 is derived, since the publicly available genome sequence of RN4220 (AFGU01000000) is not annotated. All listed genes were also present in RN4220.

[c] The predicted protein is encoded on multiple contigs. The gene name or locus tag for the predicted N-terminal is listed in the table and the remaining fragments are as follows: **MF1767 *slsA*:** AXY34_13120 (N-terminal), AXY34_13175, AXY34_13210, AXY34_13215, AXY34_13220, AXY34_13235, AXY34_13270, AXY34_13275 and AXY34_09855 (C-terminal). See Figure 2A. **MF1767 06865:** AXY34_06865 (N-terminal), AXY34_13240 and AXY34_04535 (C-terminal). **MF1862 *slsA*:** AXY37_12645 (N-terminal), AXY37_12870, AXY37_12875, AXY37_12905, AXY37_12950, AXY37_12980, AXY37_12985, AXY37_12990, AXY37_12995, AXY37_13000, AXY37_13020, AXY37_13035 and AXY37_10540 (C-terminal). See Figure 2A. **MF1862 12885:** AXY37_12885, AXY37_12900, AXY37_12975, MF1862 contig87 and AXY37_10705 (C-terminal). **MF1862 08990:** AXY37_08990 (N-terminal) and AXY37_12680 (C-terminal). **MF1844 *scsE*:** AXY36_11805 (N-terminal), AXY36_12165, AXY36_12190, AXY36_12200, AXY36_12220, AXY36_12235, AXY36_12240, AXY36_12245, AXY36_12250, AXY36_12255, AXY36_12265, AXY36_12275, AXY36_12280, AXY36_12285, AXY36_12290, AXY36_12295, AXY36_12310, AXY36_12315, AXY36_12320, AXY36_12325, AXY36_12330, AXY36_12340, AXY36_12355, AXY36_12360 and AXY36_12020 (C-terminal). See Figure 2B. **MF1844 11360:** AXY36_11360 (N-terminal) and AXY36_12105 (C-terminal). **MF1844 03795:** AXY36_03795 (N-terminal) and AXY36_06450 (C-terminal). **MF4371 *sssG*:** AXY40_12405 (N-terminal), AXY40_12590, AXY40_12620 and AXY40_12535 (C-terminal). See Figure 2D. **MF4371 *uafA*:** AXY40_12400 (C-terminal), AXY40_12580 and AXY40_05140 (N-terminal). See Figure 2C. **MF6029 *uafA*:** AXY41_11805 (C-terminal), AXY41_12195, AXY41_12225, AXY41_12230, AXY41_12235, AXY41_12245, AXY41_12280 and AXY41_05795 (N-terminal). See Figure 2C. **MF6029 *aas*:** AXY41_12165 (N-terminal), AXY41_12255 and AXY41_12140 (C-terminal). **MF1871 *embp*:** AXY38_08465 (N-terminal), AXY38_12025, AXY38_12030, and AXY38_06570 (C-terminal). **MF1871 *sdrX*:** AXY38_11695 (N-terminal) and AXY38_08460 (C-terminal). **MF1871 *sdrZL*:** AXY38_11060 (N-terminal) and AXY38_11485 (C-terminal). **MF1871 *sraP*:** AXY38_09910 (N-terminal) and AXY38_10285 (C-terminal). **MF1871 05000:** AXY38_05000 (N-terminal), AXY38_12020, and AXY38_04995 (C-terminal). **MF1871 *ssp5*:** AXY38_11615 (N-terminal) and AXY38_09585 (C-terminal). **MF1872 *sesG*:** AXY39_11895 (N-terminal) and AXY39_10235 (C-terminal). **MF1872 *sdrX*:** AXY39_11925 (N-terminal), AXY39_12020 and AXY39_10970 (C-terminal). **MF1872 *sdrZL*:** AXY39_11310 (N-terminal) and AXY39_11755 (C-terminal). **MF1872 *sraP*:** AXY39_08820 (N-terminal) and AXY39_10250 (C-terminal). **MF1872 11485:** AXY39_11485 (N-terminal) and AXY39_04020 (C-terminal). **MF1872 *ssp5*:** AXY39_11625 (N-terminal) and AXY39_11905 (C-terminal). **MF1789 *sdrE*:** AXY35_11030 (N-terminal) and AXY35_11180 (C-terminal).
